# Influence of Atomoxetine on Relationship Between ADHD Symptoms and Prefrontal Cortex Activity During Task Execution in Adult Patients

**DOI:** 10.3389/fnhum.2021.755025

**Published:** 2021-11-26

**Authors:** Atsunori Sugimoto, Yutaro Suzuki, Kiyohiro Yoshinaga, Naoki Orime, Taketsugu Hayashi, Jun Egawa, Shin Ono, Takuro Sugai, Toshiyuki Someya

**Affiliations:** ^1^Department of Community Psychiatric Medicine, Niigata University Graduate School of Medical and Dental Sciences, Niigata, Japan; ^2^Department of Psychiatry, Niigata Psychiatric Center, Nagaoka, Japan; ^3^Department of Psychiatry, Niigata University Medical and Dental Hospital, Niigata, Japan; ^4^Department of Psychiatry, Suehirobashi Hospital Keiaikai, Niigata, Japan; ^5^Department of Psychiatry, Niigata University Graduate School of Medical and Dental Sciences, Niigata, Japan; ^6^Comprehensive Medical Education Center, Niigata University School of Medicine, Niigata, Japan

**Keywords:** atomoxetine, attention-deficit/hyperactivity disorder, Conners’ adult ADHD rating scales, go/no-go task, near-infrared spectroscopy, responder group, response inhibition task

## Abstract

**Objective**: We conducted this non-randomized prospective interventional study to clarify the relationship between improved attention-deficit hyperactivity disorder (ADHD) symptoms and regional brain activity.

**Methods**: Thirty-one adult patients underwent near-infrared spectroscopy examinations during a go/no-go task, both before and 8 weeks after atomoxetine administration.

**Results**: Clinical symptoms, neuropsychological results of the go/no-go task, and bilateral lateral prefrontal activity significantly changed. A positive correlation was observed between right dorsolateral prefrontal cortex activity and Conners’ Adult ADHD Rating Scales scores. Before atomoxetine administration, no correlations between prefrontal cortex activity and clinical symptoms were observed in all cases. When participants were divided into atomoxetine-responder and non-responder groups, a positive correlation was observed between prefrontal cortex activity and clinical symptoms in the non-responder group before treatment but not in the responder group, suggesting that non-responders can activate the prefrontal cortex without atomoxetine.

**Conclusions**: Individuals with increased ADHD symptoms appear to recruit the right dorsolateral prefrontal cortex more strongly to perform the same task than those with fewer symptoms. In clinical settings, individuals with severe symptoms are often observed to perform more difficultly when performing the tasks which individuals with mild symptoms can perform easily. The atomoxetine-responder group was unable to properly activate the right dorsolateral prefrontal cortex when necessary, and the oral administration of atomoxetine enabled these patients to activate this region. In brain imaging studies of heterogeneous syndromes such as ADHD, the analytical strategy used in this study, involving drug-responsivity grouping, may effectively increase the signal-to-noise ratio.

## Introduction

Atomoxetine (ATX) is a representative drug used to treat attention-deficit hyperactivity disorder (ADHD) and is ranked as a first-line, non-stimulant treatment in national guidelines [Saito et al., 2016; Attention deficit hyperactivity disorder guideline committee of National Institute for Health and Care Excellence (NICE), 2018; Wolraich et al., 2019; Canadian ADHD Resource Alliance (CADDRA), 2020]. The use of stimulants should be carefully considered if the patient’s pre-existing condition includes a history of substance abuse or tic disorders [Canadian ADHD Resource Alliance (CADDRA), 2020], in which case treatment with non-stimulants such as atomoxetine should be considered. In addition, if stimulants cause serious cardiovascular problems or growth retardation, they must be discontinued. In such cases, atomoxetine is an important option.

Abnormalities in the prefrontal cortex (PFC), striatum, and default mode network have been identified as the neural basis of ADHD (Posner et al., 2020), and reaction suppression and other executive functions that are considered to be abnormal in ADHD individuals are mainly related to the right PFC (Fernández-Jaén et al., 2015). Because of the high density of norepinephrine transporter (NET) and low density of dopamine transporter (DAT) in the PFC, reuptake of dopamine is mainly performed *via* NET (Madras et al., 2005) and ATX is a selective norepinephrine transporter inhibitor. Animal studies have revealed that ATX increases dopamine (DA) levels in the synaptic cleft by inhibiting NET activity in PFC (Bymaster et al., 2002; Ding et al., 2014), which is considered to be the primary mechanism through which ATX improves ADHD symptoms. Although previous human studies have demonstrated reduced lateral PFC activity in ADHD patients compared with typically developing (TD) individuals (Cortese et al., 2012; Albajara Sáenz et al., 2019) and ATX administration has been shown to increase lateral PFC activity (Ota et al., 2015; Nakanishi et al., 2017; Grazioli et al., 2019), no studies have revealed an association between changes in lateral PFC activity with ATX administration and improvement in ADHD symptoms. We conducted a non-randomized prospective interventional study using near-infrared spectroscopy (NIRS) measurement in ADHD patients before and after ATX administration to clarify the relationship between improved symptoms and lateral PFC activity.

Because NIRS does not use radiation or strong magnetic fields, it has the advantage of being less invasive for some patients. In addition, whereas blood oxygen level dependent functional magnetic resonance imaging (fMRI) can only measure deoxy-Hb, NIRS has the advantage of being able to measure both oxy-Hb and deoxy-Hb. NIRS does not require the strict movement restrictions that are needed for MRI, and higher time resolution is also a benefit of NIRS measurement (Aslin and Mehler, 2005; Lloyd-Fox et al., 2010). Difficulties in measuring NIRS can occur because of spurious signals caused by slippage of the probe on the scalp, or variations in the intensity of near-infrared light at the point of contact with the scalp. However, these problems can be suppressed by improving measurement methods and analysis techniques (Aslin and Mehler, 2005). On the basis of these considerations, we decided to use NIRS in the current study.

ADHD is assumed to represent a syndrome in which multiple etiologies are superimposed (Ball et al., 2019). Therefore, diagnoses based on biomarkers and the prediction of drug reactivity are not widespread. Several previous studies have attempted to group ADHD patients according to comorbidities, resulting in new insights. A meta-analysis conducted by Cortese et al. (2012) revealed that the default mode network was included in the hypoactive region when analyzed only in ADHD patients without comorbidities. However, to the best of our knowledge, no previous functional neuroimaging studies have grouped patients by responsiveness to ADHD drug treatments to examine the pathophysiology of ADHD. Therefore, in the current study, we investigated the pathophysiology of ADHD by grouping and analyzing participants based on ATX responsivity.

## Materials and Methods

### Participants, Treatment Procedures, and Assessment of Symptoms

The participants in this study were 31 adult patients (19 male) who were diagnosed with ADHD according to The Diagnostic and Statistical Manual of Mental Disorders Fifth Edition [DSM-5; American Psychiatric Association (APA), 2013]. Participants’ ages ranged from 19 to 49 years, with a mean ± standard deviation (SD) of 31.2 ± 8.6 years. Regarding comorbid psychiatric disorders (with duplication), 10 participants had autism spectrum disorder, five had a mild intellectual disability, three had adjustment disorder, two had unspecified depressive disorder, one had a history of substance use disorder, and one had a history of child physical abuse and child neglect. All participants were confirmed to be right-handed using the Edinburgh Handedness Inventory (Oldfield, 1971). ATX treatment was started at a dose of 40 mg then increased by 40 mg every 2 weeks, with an upper limit of 120 mg, unless side-effects were detected. We used the self-reported Conners’ Adult ADHD Rating Scales (CAARS^TM^; Conners et al., 2012) to assess the clinical symptoms of all patients, both at baseline and 8 weeks after ATX treatment onset.

### Go/No-go Task

NIRS measurements were taken during a 10-minute computerized, visual-response, inhibition task, called “ADHD test program” (Norupro Light Systems Inc, 2000). In this go/no-go task, the non-target stimulus A and a target stimulus B, which closely resembled A, were randomly presented. The participant was asked to press the space key using their index finger as quickly as possible when A was presented (response) and to refrain from pressing the space key when B was presented (response inhibition). Using the preset “Adult Standard 2” setting. The division area was set to five, the screen was divided into 5 × 5 = 25 squares, and targets appeared randomly at any position. The target appearance time was 200 ms, the interval wait time was 1,300 ms and the interval time randomization rate was 50%. With an interval wait time of 1,300 ms and an interval time randomization rate of 50%, the time to the presentation of the next stimulus varied between 650 and 1, 950 ms. Therefore, the number of trials fluctuated slightly each time. However, because the target appearance time was 200 ms and the standard sensory standby time was 1,300 ms, the number of trials for 10 min converged at approximately 400 times. The target presentation rate was 50%, and the probability that stimulus B was presented was 50%. In most cases, go/no-go tasks have a target presentation rate of approximately 20%, but this high target presentation rate characterized our task. The screen used for stimulus presentation was 17 inches in size (33.7 cm × 27.0 cm), and the positions of the participant and the screen were adjusted to maintain a distance of 50 cm between the screen and the participants’ eyes. To measure the Δ[Oxy-Hb] values purely associated with executing the reaction inhibition task, and to remove background elements, such as motion planning or motion starting, 10 s of pre- and post-task periods were provided. During the pre- and post-task periods, participants were asked to tap the desk iteratively with their index finger, using a motion that was equivalent to pressing the space key.

### NIRS Data Acquisition

**Figure 1:**
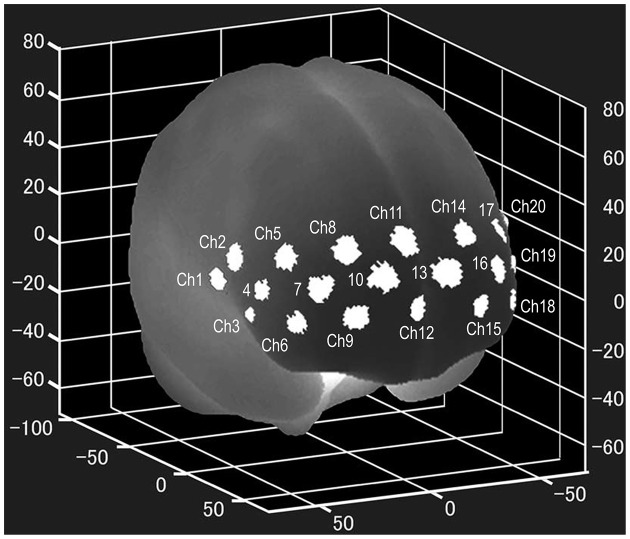
Locations of the NIRS channels. The mean estimated locations of the studied channels of the wearable 16 Ch-NIRS WOT-100 system (HITACHI, Tokyo, Japan), represented in the MNI standard brain space, using the probabilistic estimation method for 10 volunteers (Atsumori et al., 2010). Ch 20, 21, and 22 are on the left side of the brain and are difficult to see in the figure. Ch 1, 2, 3, 20, 21, and 22 are optional channels, which our institution does not have access to. NIRS, near-infrared spectroscopy; MNI, Montreal Neurological Institute.

NIRS examinations were performed using a wearable 16 Ch-NIRS WOT-100 system (HITACHI, Tokyo, Japan) before and 8 weeks after the onset of ATX administration. All participants underwent two NIRS measurements. In pretreatment measurements, participants had never taken ATX before. In the post-treatment measurement, NIRS measurement was performed 12 h or more after the last ATX administration. NIRS measures changes in oxygenated hemoglobin levels (Δ[Oxy-Hb]) in the PFC, using near-infrared rays, and can evaluate activity during task execution. The location of each channel was estimated using the probabilistic estimation method (Singh et al., 2005; Atsumori et al., 2010) in the Montreal Neurological Institute (MNI) standard brain space, as shown in Figure 1. The sampling rate was set to 5 Hz, and baseline correction was performed using linear fitting based on two points of the pre- and post-task period. The pre-task baseline used for the baseline correction was the last point of the 10-s pre-task period, and the post-task baseline was the last point of the post-task period. As the activation value, we used the average time series data with baseline correction for the entire measurement period during task execution. Microsoft Excel was used for baseline correction and calculation of activation values.

### Statistical Analysis

IBM SPSS Statistics 24 (IBM Japan, Tokyo, Japan) was used for all statistical analyses. A 10-min average of changes in Δ[Oxy-Hb] for each channel during the task was calculated, and prefrontal cortex activity was examined to detect changes after ATX administration. Then, for channels in which activity changed after ATX administration, the relationship between activity at that site and clinical symptoms evaluated by CAARS was examined. Paired Student’s t-tests were used to test the difference, Pearson’s correlation coefficient test was used to test the correlation, and the significance level was set to 5%. The Bonferroni correction was used to correct for multiple testing. However, in consideration of the criticism that the broad application of the Bonferroni correction is overly strict, the correction was separately adapted into four categories: each item of CAARS, task performance, changes in Δ[Oxy-Hb], and correlation between PFC activity and CAARS items. The change was calculated as “post–pre,” and if the value decreased after treatment, the change was negative.

## Results

The final ATX doses ranged from 25 to 120 mg, with a mean ± SD of 95.3 ± 34.5 mg. The CAARS scores before and after administration are shown in Table 1, and all scores other than those for item D (Problems with self-concept) significantly improved after ATX administration. Results of the ADHD test program are also shown in Table 1. All indices except mean reaction time significantly improved after ATX administration. The Δ[Oxy-Hb] values before and after ATX administration are shown in Table 2, and activity changed in bilateral lateral PFC (Ch 5, 6, 17, 18).

**Table 1:** Scores of Conners’ Adult ADHD Rating Scales and ADHD test program.

	Baseline	8 week	*p* value
Conners’ Adult ADHD Rating Scales (CAARS^TM^)
A: Inattention/Memory Problems	79.0 ± 9.0	72.2 ± 10.0	0.000075*
B: Hyperactivity/Restlessness	68.6 ± 11.8	65.1 ± 11.6	0.025*
C: Impulsivity/Emotional Lability	70.2 ± 12.9	64.6 ± 12.0	0.013*
D: Problems with Self-Concept	67.1 ± 8.2	66.3 ± 9.2	0.548
E: DSM-IV Inattentive Symptoms	81.9 ± 9.3	73.8 ± 10.4	0.000054*
F: DSM-IV Hyperactive-Impulsive	74.4 ± 12.6	66.1 ± 13.0	0.001*
G: DSM-IV ADHD Symptoms Total	81.4 ± 8.4	72.5 ± 10.2	0.000096*
H: ADHD Index	76.0 ± 7.8	71.2 ± 8.9	0.005*
ADHD test program (NoruPro Light Systems Inc.)
Correct answer rate (%)	92.7 ± 6.2	95.3 ± 5.3	0.000179*
SD of correct answer rate	3.72 ± 1.74	2.96 ± 1.85	0.007*
Mean reaction time (ms)	494.0 ± 53.7	495.2 ± 50.9	0.877
SD of reaction time	76.3 ± 19.4	66.9 ± 15.6	0.001*
Omission error (%)	0.15 ± 0.03	0.13 ± 0.03	0.000019*
Commission error (%)	3.75 ± 5.54	2.35 ± 4.06	0.008*

Each score is expressed as mean ± SD; *‥. <0.05. ADHD, attention-deficit hyperactivity disorder; DSM, Diagnostic and Statistical Manual of Mental Disorders.

**Table 2:** Prefrontal activity as Δ[Oxy-Hb] measured by near-infrared spectroscopy.

	Baseline	8 week	*p*			Baseline	8 week	*p*
Ch4	−0.05 ± 0.20	−0.03 ± 0.30	0.621		Ch12	0.01 ± 0.20	−0.02 ± 0.22	0.549
Ch5	0.28 ± 0.15	0.83 ± 0.13	0.016*		Ch13	0.03 ± 0.17	0.01 ± 0.27	0.737
Ch6	−0.02 ± 0.16	0.05 ± 0.15	0.039*		Ch14	0.03 ± 0.14	0.05 ± 0.12	0.405
Ch7	−0.05 ± 0.32	0.05 ± 0.22	0.137		Ch15	−0.03 ± 0.18	0.03 ± 0.17	0.112
Ch8	0.02 ± 0.13	0.06 ± 0.19	0.374		Ch16	0.01 ± 0.14	0.09 ± 0.21	0.070
Ch9	−0.002 ± 0.18	0.02 ± 0.17	0.590		Ch17	0.01 ± 0.19	0.08 ± 0.12	0.031*
Ch10	−0.12 ± 0.51	0.06 ± 0.27	0.138		Ch18	−0.0004 ± 0.11	0.08 ± 0.16	0.015*
Ch11	0.04 ± 0.13	0.01 ± 0.20	0.567		Ch19	0.03 ± 0.14	0.06 ± 0.24	0.588

Each score is expressed as mean ± SD; *… <0.05.

**Figure 2:**
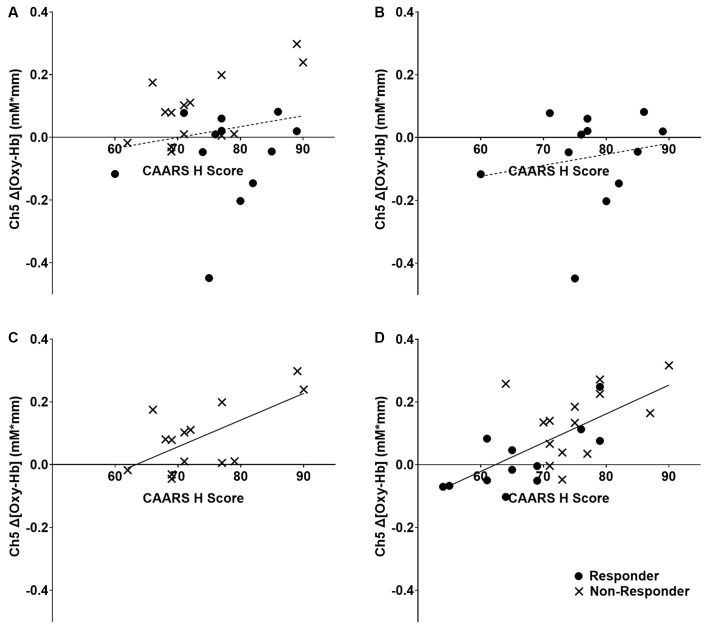
Relationship between right dorsolateral prefrontal cortex activity and clinical ADHD symptoms. The dashed line indicates that there is no significant correlation, and the solid line indicates that there is a significant correlation. The regression lines refer to all individuals in the panel, including both responder and non-responder groups. **(A)** All participants at baseline, *r* = 0.129, *p* = 0.522. **(B)** ATX-responder group at baseline, *r* = 0.179, *p* = 0.579. **(C)** ATX non-responder group at baseline, *r* = 0.646, *p* = 0.013. **(D)** All participants at 8 weeks, *r* = 0.694, *p* < 0.001. ADHD,attention-deficit hyperactivity disorder; ATX, Atomoxetine.

Among the four channels, only Δ[Oxy-Hb] in the right dorsolateral PFC (Ch 5) showed positive correlations with the CAARS items D, F, G, and H of CAARS after ATX administration (*r* = 0.464, 0.430, 0.473, and 0.694, respectively; *p* = 0.020, 0.032, 0.017, and <0.001, respectively), and significant correlations were not observed for the other channels. Because the CAARS item H showed the strongest correlation with Ch5, item H was used to distinguish responders. The mean change in H-score after ATX administration was −4.19, the SD was 8.22, and the median was −2. The distribution of changes in item H exhibited a clear bimodality between participants exhibiting an improvement of 6 points or more and those exhibiting an improvement of 2 or less (including no change and deterioration). No participants exhibited H-score changes of −3, −4, or −5. On the basis of these findings, we defined participants with an H-score improvement of 4 or more after ATX administration as the responder group, and those with an improvement of 3 or less (unchanged or worse) as the non-responder group. As shown in Figure 2, no correlation was found between Δ[Oxy-Hb] values and H-score before ATX administration in the responder group, whereas in the non-responder group, a positive correlation was observed.

Regarding the correlation between dose and changes in symptoms and PFC activity, in all participants, there were no correlations between the dose, change in item H, and change in Ch5 (*r* = 0.252, 0.246; *p* = 0.214, 0.182). When only responders were analyzed, there were no correlations between the dose, the change in item H, and the change in Ch5 (*r* = 0.443, 0.334; *p* = 0.149, 0.273).

Regarding the correction for multiple tests, even after Bonferroni correction, significant differences were found in CAARS items A, E, F, G, and H for symptom improvement (*p* < 0.00625), and in the correct answer rate, SD of the correct answer rate, SD of reaction time, omission errors, and commission errors in task performance (*p* < 0.00833). Although the increase in Δ[Oxy-Hb] in the lateral PFC after ATX administration could not be maintained after correction (*p* < 0.0125), a significant correlation between Ch 5 and item H was maintained for the correlations between Ch 5, 6, 17, 18 and CAARS items A, B, C, E, F, G, H (*p* < 0.00179).

## Discussion

### Relationship Between ADHD Symptoms and Prefrontal Cortex Activity

The results of this study suggested that ATX administration increased lateral PFC activity, indicating that right dorsolateral PFC (DLPFC) activity may be related to clinical ADHD symptoms. Although previous studies have demonstrated reduced lateral PFC activity in ADHD patients compared with TD individuals (Cortese et al., 2012; Albajara Sáenz et al., 2019) and ATX administration has been shown to increase lateral PFC activity (Ota et al., 2015; Nakanishi et al., 2017; Grazioli et al., 2019), the current study clarified the relationship between ATX-induced change in right DLPFC activity and clinical ADHD symptoms. The positive correlation observed between right DLPFC activity and each CAARS item did not allow conclusions about causality. However, pathologically, given that brain activity is always unidirectional in causing symptoms, it is likely that ADHD symptoms were more severe in individuals with more intense PFC activity when performing the same task. In clinical practice, it is often observed that people with severe symptoms need to mobilize more concentration to perform tasks that people with mild symptoms can easily perform. This positive correlation suggests that all participants in both groups exhibited the right DLPFC activity that was correlated with symptoms during task performance after ATX administration. However, before ATX administration, a correlation was observed between right DLPFC activity and ADHD symptoms in the non-responder group, whereas no similar correlation was observed in the responder group. These findings suggest that the non-responder group showed right DLPFC activity that was correlated with symptoms during task performance even before ATX administration, whereas individuals in the responder group did not show similar activity before treatment, and the same site showed symptom-correlated activity only after treatment. These mechanisms can explain the improvement of ADHD symptoms by ATX administration and have important implications for understanding brain local drug reactions that bridge the molecular-level mechanisms (Bymaster et al., 2002; Ding et al., 2014) and symptom-level findings of previous studies.

It is necessary to consider the mechanisms underlying the strong correlation between ADHD symptoms and right DLPFC activity, and the lack of a correlation between ATX dose and changes in symptoms or changes in right DLPFC activity. The level of symptoms exhibited by the responders who received ATX and the non-responders who did not receive ATX, and the extent of right DLPFC activation during task performance (both of which were correlated) appeared to be defined by some other factor. This factor may be related to features such as the striatum, cerebellum, and the broader default mode network. In other words, although ATX may provide a way of releasing suppression or mask, the degree of symptoms of responders after releasing suppression or mask appears to be defined by some other factor. Thus, the effect of releasing suppression or mask on an individual’s symptoms (i.e., how much the symptoms apparently change because of treatment) is not correlated with the dose of ATX.

Although Schulz et al. (2012) reported that increased right inferior frontal gyrus activity was significantly associated with improved ADHD symptoms following ATX treatment, no study has reported a similar correlation for right DLPFC activity. Both the inferior frontal gyrus and the DLPFC, especially in the right hemisphere, are involved in response inhibition functions (Garavan et al., 2006). However, the DLPFC is associated with “selecting” the inhibitory response, whereas the inferior frontal gyrus is associated with “inhibiting” the response. We used a task involving a high rate of target stimuli and a low commission error rate (Norupro Light Systems Inc, 2000), whereas Schulz et al. (2012) used a task with a low rate of target stimuli and a high commission error rate (Durston et al., 2002). When the appearance rate of target stimuli is low, the factor that “inhibits” the response is strengthened, and when the appearance rate of target stimuli is high, the factor that “selects” response inhibition is strengthened. Therefore, these differences in tasks may explain the differences in results between the two studies. Given the above points, the importance of task selection should be examined in more depth in future functional brain imaging studies, including fMRI and fNIRS studies.

In the current results, CAARS items A, B, C, E, F, G, and H were significantly improved, but item D was not significantly changed. For items A, B, C, E, F, G, and H, which are the core symptoms of ADHD, ATX administration has a direct effect, and it is possible that the symptoms improved relatively early. Although the observation period for this study was 8 weeks, if there is any improvement in item D (which indicates problems with self-concept), it may appear later.

### Grouping by Drug Responsivity

Whereas previous studies did not separately examine drug-responder and non-responder groups, the adoption of this grouping method in the current study may have successfully clarified the mechanisms of symptom improvement induced by ATX. Although Cortese et al. (2012) divided ADHD individuals into several groups based on comorbidities in their meta-analysis of fMRI studies, which provided new insight associated with functional imaging of ADHD patient brains, grouping by drug reactivity should also be considered. In functional brain imaging studies and genetic studies, if patients are a heterogeneous population with multiple pathologies, the signal-to-noise ratio cannot be effectively increased simply by increasing the size of samples, such as by performing meta-analyses. Rather, the signal-to-noise ratio must be increased by extracting and analyzing specific and uniform groups of patients. The division of ADHD patients into drug-responder and non-responder groups represents a reasonable approach that should be applied in future functional brain imaging and genetic studies. However, although the analyses of this study were successfully performed following the classification based on the CAARS score for convenience, the actual patient population remains a spectral aggregate. Future researchers should consider this point, even when stratifying patients into two or more categories.

### Limitations

An important limitation is that this study was not a randomized controlled trial. Furthermore, because CAARS was self-assessed, expectancy effects cannot be ruled out, particularly for CAARS changes after ATX administration. However, we believe that the changes in task performance and the statistical robustness of the correlation between right DLPFC activity and symptoms enabled the current study to overcome some of the limitations of previous studies.

In this study, we did not include a TD group as a control group. Assuming that the ATX non-responder group exhibits ADHD symptoms because of a condition other than the impairment of PFC function, a TD group would be expected to exhibit similar distribution on the y-axis and left side distribution on the x-axis than the non-responder group in the graph shown in Figure 2. If this prediction is correct, the whole ADHD group, including both the ATX-responder and non-responder groups, would be expected to have lower y-axis values than the TD group, which would be consistent with the observations in previous studies showing reduced PFC activity in ADHD patients compared with that in the TD group (Albajara Sáenz et al., 2019). Further research should examine differences in regional brain function between an ATX-responder group, a non-responder group, and a TD group.

The problem of multiple testing is considered to be an important limitation of this study. Although most of the main results of this study were maintained after correction for multiple testing, the increase in Δ[Oxy-Hb] after ATX administration was not maintained after Bonferroni correction. We carefully considered this point before interpreting and considering the results, and readers should take this issue into account when interpreting the current findings.

Because of the wide age range in the sample (19–49 years) in the current study, there may have been age-related variability in task performance and PFC activity. Although the lack of a method for controlling for the effect of age on task performance and PFC activity is a limitation of this study, it should be noted that there was no significant correlation between age and task performance or PFC activity.

In the current study, we found no associations between clinical symptoms and task performance. The lack of a correlation between neuropsychological task performance and the self-reported symptom scale has been previously reported for ADHD patients (Toplak et al., 2013). Leontyev et al. (2018) performed a dynamic assessment and argued that inattention or hyperactivity in ADHD patients appears during the process of deciding the optimized final choice (movement of the mouse cursor up to that point) and during unpurposive behavior in which the participant self-decided their conduct, which cannot be measured when only considering the optimized final selection in a time-limited environment. The go/no-go task used in this study only measured the optimized final selection, potentially explaining why no correlation was observed between task performance and clinical symptoms. Leontyev et al. (2018) proposed a go/no-go task that traces the movement of the mouse cursor until the final selection is made, as a countermeasure. Such an approach may be necessary to detect associations between clinical symptoms and task performance. However, a previous study (Yasuhara, 2006) clarified the differences between the ADHD group and the control group, and, in this study as well, the parameters changed significantly after ATX administration, confirming a robust correlation between brain activity and symptoms during task execution. On the basis of these factors, we believe that this task was appropriate for addressing our research questions.

Previous studies (Ishii-Takahashi et al., 2015; Kim et al., 2015; Schulz et al., 2017) have attempted to predict responses to drug treatments before administration, based on functional brain imaging findings. However, as shown in Figure 2, the distribution of responders and non-responders before ATX administration overlapped, and the clinical application of predicted effectiveness appears to be relatively difficult. However, approaches that use machine learning are promising for predicting drug responses (Kim et al., 2015). Further considerations of multiple etiologies, such as those described above, by performing comprehensive examinations of differential activities in multiple regions of interest, may be necessary to predict drug responsiveness using brain functional imaging findings.

## Data Availability Statement

The datasets presented in this article are not readily available because the datasets generated and/or analyzed during the current study are not publicly available due to lack of ethics committee permission and not having been part of the consent process. The reasonable request will be raised with Niigata University Genetic Ethics Review Committee. Requests to access the datasets should be directed to ethics@adm.niigata-u.ac.jp.

## Ethics Statement

The studies involving human participants were reviewed and approved by Genetic Ethics Review Committee of Niigata University School of Medicine. The patients/participants provided their written informed consent to participate in this study.

## Author Contributions

AS: conceptualization, methodology, validation, investigation, data curation, formal analysis, writing—original draft, writing—review and editing, project administration, and funding acquisition. YS: conceptualization, methodology, validation, formal analysis, writing—review and editing, and project administration. KY, NO, and TH: investigation and resources. JE: methodology, validation, and writing—review and editing. SO and TSu: writing—review and editing, funding acquisition. TSo: methodology, validation, formal analysis, writing—review and editing, supervision, and project administration.

## Conflict of Interest

The authors declare no conflicts of interest with respect to this study, except for the following financing issues. AS and SO have received contract research funds from the Hospitals Bureau Niigata Prefecture Government. YS has received research support or honoraria from Janssen Pharmaceutical K.K., Mitsubishi Tanabe Pharma Co. Ltd., and Otsuka Pharmaceutical Co. Ltd. SO and TSu have received Grant-in-Aid for Scientific Research (KAKENHI) from Japan Society for the Promotion of Science. TSo has received research support or honoraria from Asahi Kasei Pharma Corp., Astellas Pharma Inc., Daiichi Sankyo Co. Ltd., Dainippon Sumitomo Pharma Co. Ltd., Eisai Co. Ltd., Eli Lilly Japan K.K., GlaxoSmithKline K.K., Janssen Pharmaceutical K.K., Meiji Seika Pharma Co. Ltd., Mitsubishi Tanabe Pharma Co. Ltd., Mochida Pharmaceutical Co. Ltd., MSD K.K., Otsuka Pharmaceutical Co. Ltd., Pfizer Japan Inc., Shionogi & Co. Ltd., Tsumura & Co., and Yoshitomi Pharmaceutical Industries. The other authors have received no specific grants from any funding agency in the public, commercial, or not-for-profit sectors for this research. The funders had no role in study design, data collection and analysis, decision to publish, or preparation of the manuscript. The remaining authors declare that the research was conducted in the absence of any commercial or financial relationships that could be construed as a potential conflict of interest.

## Publisher’s Note

All claims expressed in this article are solely those of the authors and do not necessarily represent those of their affiliated organizations, or those of the publisher, the editors and the reviewers. Any product that may be evaluated in this article, or claim that may be made by its manufacturer, is not guaranteed or endorsed by the publisher.
